# Mitochondrial Breast Cancer Resistant Protein Sustains the Proliferation and Survival of Drug-Resistant Breast Cancer Cells by Regulating Intracellular Reactive Oxygen Species

**DOI:** 10.3389/fcell.2021.719209

**Published:** 2021-09-28

**Authors:** He Zhang, Xingxing Han, Zhaosong Wang, Zhiyong Wang, Yanfen Cui, Ran Tian, Yuying Zhu, Baoai Han, Hui Liu, Xiaoyan Zuo, Sixin Ren, Jianfei Tian, Ruifang Niu, Fei Zhang

**Affiliations:** ^1^Public Laboratory, National Clinical Research Center for Cancer, Tianjin Medical University Cancer Institute and Hospital, Tianjin, China; ^2^Key Laboratory of Cancer Prevention and Therapy, Tianjin, China; ^3^Tianjin’s Clinical Research Center for Cancer, Tianjin, China; ^4^Key Laboratory of Breast Cancer Prevention and Therapy, Tianjin Medical University, Ministry of Education, Tianjin, China

**Keywords:** drug-resistant breast cancer, breast cancer resistant protein, reactive oxygen species, survival, proliferation

## Abstract

ATP-binding cassette (ABC) transporter family are major contributors to the drug resistance establishment of breast cancer cells. Breast cancer resistant protein (BCRP), one of the ABC transporters, has long been recognized as a pump that effluxes the therapeutic drugs against the concentration gradient. However, recent studies suggest that the biological function of BCRP is not limited in its drug pump activity. Herein, the role of BCRP in the proliferation and survival of drug-resistant breast cancer cells was investigated. We found that BCRP is not the major drug pump to efflux epirubicin in the resistant cells that express multiple ABC transporters. Silencing of BCRP significantly impairs cell proliferation and induces apoptosis of the resistant cells *in vitro* and *in vivo*. RNA-sequencing and high-throughput proteomics suggest that BCRP is an inhibitory factor of oxidative phosphorylation (OXPHOS). Further research suggests that BCRP is localized in the mitochondria of the resistant cells. Knockdown of BCRP elevated the intracellular reactive oxygen species level and eventually promotes the cell to undergo apoptosis. This study demonstrated that BCRP exerts important onco-promoting functions in the drug-resistant breast cancer cells independent of its well-recognized drug efflux activity, which shed new light on understanding the complex functional role of ABC transporters in drug-resistant cells.

## Introduction

Breast cancer is one of the major malignant tumors in women, accounting for 30% of all newly diagnosed cancers in women worldwide (Sung et al., 2021). Chemotherapy plays an important role in the management of breast cancer; however, cancer cells always develop drug-resistance ability, which greatly limits the outcome of chemotherapeutic treatment (Fletcher et al., 2016). The establishment of the drug resistance ability of cancer cells is a long-term multistage process that is accompanied by complex reprogramming. Cells always acquire a strong ability to export the chemotherapy drugs, thereby increasing the difficulty of their elimination by treatments (Holohan et al., 2013; Kathawala et al., 2015; Fletcher et al., 2016). Moreover, drug-resistant cells establish strong capabilities to survive, even in the fluctuated environment in which they are not favored (Bukowski et al., 2020). Therefore, understanding the molecular foundation that supports the enhanced survival phenotype of drug-resistant cells is needed.

The ATP-binding cassette (ABC) transporter family comprises 48 identified members in humans to date. They mediate the active transport of specific substrates by consuming energy from ATP (Fletcher et al., 2016). Several of them, such as P-glycoprotein (P-gp), multidrug resistance protein 1 (MRP1), and breast cancer resistant protein (BCRP), are highly expressed in drug-resistant cancer cells to mediate the efflux of drugs, thereby conferring drug resistance ability to cancer cells (Mao and Unadkat, 2015; Domenichini et al., 2019; Muriithi et al., 2020). Notably, ABC transporters are involved in a broad range of physiological processes that do not completely depend on its transport activity, but the related studies are still limited.

BCRP (encoded by *ABCG2* gene) is a well-characterized drug pump generally believed to be localized in the cell membrane to mediate the efflux of chemotherapeutic drugs (Mao and Unadkat, 2015; Taylor et al., 2017). High expression of BCRP was linked with a worse prognosis of many types of cancer, including leukemia and breast cancer (Fletcher et al., 2016; Muriithi et al., 2020). Recent studies have suggested that BCRP can also be located in the mitochondria, nucleus, and cytoplasm of cells (Bhatia et al., 2012; Liang et al., 2015). It has been demonstrated that BCRP can be translocated into cell nuclei to regulate the activity of *CDH1* promoter in lung cancer (Liang et al., 2015). Other reports have suggested that BCRP knockdown inhibits proliferation in DU145 and MCF7 cells by regulating cell cycle progression (Chen et al., 2010). BCRP has also been identified as a marker for side-population cells that exert stem-cell-like features (Singh et al., 2010; Westover and Li, 2015). These findings suggest that BCRP may play important functional roles in cancer cells independently of its drug pump activity; however, the detailed mechanism that is responsible for these drug-pump-independent roles of BCRP in various malignancies is poorly investigated.

Mitochondria are the “powerhouse” of eukaryotic cells and enclose the key enzymes for oxidative phosphorylation (OXPHOS) and ATP synthesis (Okon and Zou, 2015). In addition to providing power to the cell, several important biological processes can be linked to mitochondria, such as producing reactive oxygen species (ROS), synthesizing essential metabolites for cell proliferation, and regulating signaling transduction in cells (Abate et al., 2020; Bock and Tait, 2020). Mitochondria also confer strong flexibility of proliferation and survival to cells by regulating specific metabolic processes (Vyas et al., 2016). Several ABC transporters such as ABCB7, ABCB10, ABCB8, ABCB6, and ABCG2 have been found localized in mitochondria (Zutz et al., 2009). ABCB7 has been demonstrated to regulate apoptotic and non-apoptotic cell death by modulating mitochondrial ROS and HIF1α-driven NFκB signaling (Kim et al., 2020). BCRP can also be localized in the mitochondria of drug-resistant breast cancer cells to mediate the transport of specific substrates (Solazzo et al., 2009). However, whether BCRP is involved in the regulation of mitochondria function and cell proliferation and survival remains unknown.

In the present study, we took advantage of previously established drug-resistant breast cancer cell lines and investigated the biological function of mitochondrial BCRP in drug-resistant breast cancer cells. We found that BCRP was localized in mitochondria. RNA-sequencing (RNA-seq) and proteomic analysis suggested that BCRP suppressed OXPHOS to maintain the homeostasis of intracellular ROS level. Notably, BCRP silencing drastically elevated the intracellular ROS level and ultimately promoted cell apoptosis. This study demonstrated that mitochondrial BCRP was essential for drug-resistant breast cancer cells to sustain their proliferation and antiapoptosis ability.

## Materials and Methods

### Cell Culture

Human breast cancer cell lines MDA-MB-468 (MDA-468), BT549, and SK-BR-3 were purchased from the American Type Culture Collection (ATCC, Manassas, VA, United States). The epirubicin (EPI)-resistant cell lines were established based on MDA-468 and SK-BR-3 cells by long-time multistage induction using an increasing concentration of EPI, as previously described, named as MDA-MB-468/EPI (468/EPI) and SK-BR-3/EPI (SK/EPI) (Zhang et al., 2015b; Fan et al., 2019). HEK293T cell line was purchased from ATCC. MDA-468 and 468/EPI cells were maintained in DME/F12 medium (Hyclone, South Logan, UT, United States), SK-BR-3 and SK/EPI cells were cultured in RPMI-1640 medium (Hyclone), HEK293T cells were cultured in Dulbecco’s modified Eagle’s medium (DMEM) medium, and all media were supplemented with 10% fetal bovine serum (HyClone), 100 U/ml penicillin, and streptomycin. The cells were cultured in a humidified incubator containing 5% CO_2_ at 37°C. All cell lines used in this study were routinely checked by morphological observation and tested free from mycoplasma contamination. The mycoplasma contamination was tested using Myco-Blue Mycoplasma Detector (Vazyme, China) following the instructions of the manufacturer.

### Plasmid Construction, Lentivirus Production, and Stable Cell Lines Generation

The BCRP overexpression vector pCDH-BCRP was established by insertion of BCRP open reading frame (amplified using primers BCRP-CE-F and BCRP-CE-R) into pCDH backbone at *Xbal*I and *BamH*I by using ClonExpress Ultra One Step Cloning Kit (Vazym, China). The doxycycline (Dox) inducible BCRP knockdown vectors EZ-tet-shBCRP and EZ-tet-shControl were established by annealing DNA oligos (shBCRP-#1, #2-F/R, and shControl-F/R) and then inserting into EZ-tet-pLKO backbone at *Nhe*I and *EcoR*I. All vectors were verified by Sanger sequencing. The DNA oligos used in plasmid construction as shown in Supplementary Table 2.

The lentivirus was produced by a standard three-plasmid system. Briefly, psPAX2 7.5 μg, pMD2.G 2 μg, and 10 μg lentiviral vectors (EZ-tet-shBCRP or EZ-tet-shControl) were cotransfected into HEK293T cells using PEI reagent, and then the viral-containing medium was collected 48 h after transfection.

The Dox inducible BCRP knockdown cell line 468/EPI-shBCRP-tet and the control cell line 468/EPI-shControl-tet were established by incubating 468/EPI cells in the medium consisting of the viral-containing medium and complete growth medium at 1:1 ratio for 72 h, and then the infected cells were screened by hygomycin at 200 μg/ml for 2 weeks.

### Transfection

The stealth small interference RNAs (siRNAs) that specifically target BCRP or P-gp mRNA were synthesized by Thermal Fisher Scientific (Waltham, MA, United States). The siRNAs were transfected into the cells by using lipofectamine RNAiMax (Thermal Fisher Scientific) following the manufacturer’s instructions. The sequence of siRNAs used in this study is shown in Supplementary Table 2.

### Western Blot Analysis

The detailed procedure for Western blot is described previously (Zhang et al., 2015a). In brief, the cells were lyzed by 1 × SDS lysis buffer, then 20–50 μg protein was loaded into polyacrylamide gels following transfer and blocking. The primary and secondary antibodies used in this study are shown in Supplementary Table 1. The protein signal was detected by a chemiluminescence system using ECL (Millipore, Billerica, MA, United States).

### Quantitative Real-Time PCR Analysis

The detailed method for carryout quantitative real-time PCR (qRT-PCR) is previously described (Zhang et al., 2020). Briefly, cells were lyzed using TRIzol reagent (Life Technologies, Carlsbad, CA, United States), and the RNA was isolated and reverse transcribed into cDNA. All qRT-PCRs were run in triplicate and normalized with the β-actin housekeeping gene. The primers for qRT-PCR experiments are shown in Supplementary Table 2.

### Cell Proliferation Assay and Colony Formation Assay

Cell proliferation was determined by cell count kit 8 (CCK8) assay as previously described (Yuan et al., 2020). The SK/EPI and 468/EPI cells were seeded into 96-well plates in triplicate at a density of 3 × 10^3^–1 × 10^3^. To determine the relative cell number, 100 μl of CCK8 reagent was added into each well and incubated at 37°C for 2–3 h, and then the absorbance was measured at 450 nm.

For colony formation assay, 2,000–800 cells were seeded into a 3.5-mm dish and cultured in complete growth medium for 2 (468/EPI) or 3 weeks (SK/EPI). Then, the cells were washed with ice-cold PBS, fixed by methanol, and stained using crystal violet staining buffer.

### Mitochondria Isolation

Mitochondrial isolation was performed using Mitochondrial Extraction Kit (Solarbio, China) following the protocol of the manufacturer. Approximately 5 × 10^7^ cells were cultured and collected for the mitochondrial extraction procedure. The collected cells were gently homogenized by lysis buffer using a glass homogenizer and then underwent a series of differential centrifugation to separate the pure mitochondria. Then, the purified mitochondria were resuspended in 50 μl store buffer.

### Immunofluorescence and Terminal Deoxynucleotidyl Transferase Biotin-dUTP Nick End Labeling Staining

For immunofluorescence (IF) staining, the cells were seeded in 12-well plates preplaced with a polylysine-coated coverglass and incubated for 24 h. Then, the cells were incubated with MitoTracker (Beyotime, China) working solution (diluted by complete growth medium at a ratio of 1:1,000) and incubated at 37°C for 20 min. Then, the cells were washed by PBS, fixed with 4% paraformaldehyde, permeabilized by 0.1% Triton X-100, and blocked by 3% BSA, following incubation with primary and fluorophore-labeled secondary antibodies. Then, the stained cells were imaged by a confocal microscope. The IF images were processed and analyzed by ImageJ to investigate the colocalization between BCRP and mitochondria.

For transferase biotin-dUTP nick end labeling (TUNEL) staining, the tumor tissues were fixed by 4% paraformaldehyde and embedded by paraffin, and then, 5-μm slides were made. Then, the TUNEL staining was carried out using a TUNEL staining kit (Beyotime) following the instructions of the manufacturer.

### RNA Sequencing, Proteomic Analysis, and Bioinformatics

For transcriptome RNA-seq, SK/EPI, SK-BR-3, SK/EPI-siBCRP, and SK/EPI-siControl cells were prepared in triplicates and lysed by TRIzol reagent (Invitrogen). Then the cell lysates were processed and sequence by NovoGene (Beijing, China). For high-throughput proteomics analysis, approximately 1 × 10^7^ SK/EPI and SK-BR-3 cells were prepared in triplicates, then the cells were processed by GeneChem (Beijing, China) and analyzed by Tandem Mass Tags (TMT) based proteomics.

The gene set enrichment analysis (GSEA) and was performed using WebGestalt database^1^ using differentially expressed proteins and mRNAs in the proteomic and RNA-seq data.

### Intercellular Reactive Oxygen Species Measurement

The intercellular ROS was analyzed using the ROS Assay Kit (Beyotime, China) following the protocol of the manufacturer. The cells were seeded in six-well plates and treated accordingly, and then washed by PBS and incubated with DCFH-DA ROS probe (diluted in culture medium at a ratio of 1:1,000) at 37°C for 20 min, and then the cells were trypsinized and washed. Then, the stained cells were analyzed using a follow cytometer using the fluorescein isothiocyanate (FITC) channel.

### Cell Apoptosis Analysis

The cell apoptosis analysis was carried out using the Annexin V–FITC/propidium iodide (PI) Apoptosis Detection Kit (Vazyme, China) following the instruction of the manufacturer. The cells were seeded in six-well plates and treated accordingly, then trypsinized, washed by PBS, and resuspended in 300 μl binding buffer. The cell suspension was then incubated with 5 μl Annexin V–FITC and 5 μl PI staining reagent at 25°C in the dark for 10 min, and then, the stained cells were analyzed by a follow cytometer using the FITC and PE channels.

### Animal Experiments

Four- to five-week-old of female BALB/-nude mice were purchased from Beijing Biotechnology Co., Ltd. (Bejing, China). Approximately 5 × 10^7^ Dox inducible BCRP knockdown cells SK/EPI-shBCRP-tet, SK/EPI-shControl-tet, 468/EPI-shBCRP-tet, or 468/EPI-shControl-tet were inoculated in the mice subcutaneously. Then, the mice were gavaged using 40 mg/kg Dox or H_2_O every 2 days 14 days after inoculation. The volume of tumors in the mice was measured every 2 days, and then, mice were sacrificed 22 days after gavage. Then, the tumors were dissected, washed, imaged, and fixed. All experimental operations comply with the requirements of the Animal Ethics Committee of Tianjin Medical University Cancer Institute and Hospital.

### Statistical Analysis

All data were analyzed using Prism 8 (GraphPad). Unpaired Student’s *t*-test and one-way or two-way ANOVA test (followed by Dunnett’s *post hoc* tests) were used to compare between different groups for statistical significance, and *p* < 0.05 was considered statistically significant. All the data were presented as mean ± SD.

## Results

### Breast Cancer Resistant Protein Is Not the Dominant Efflux Pump in Drug-Resistant Cells With Multiple Activated ATP-Binding Cassette Transporters

**FIGURE 1:**
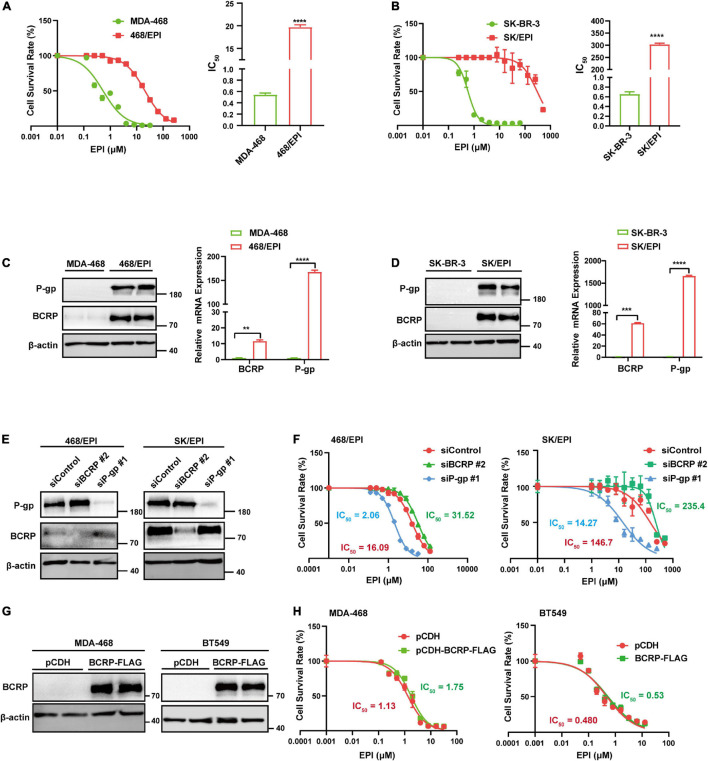
BCRP is not the dominant efflux pump in drug-resistant cells with multiple activated ABC transporters. **(A)** Dose–response curve of MDA-MB-468 (MDA-468) and MDA-MB-468/EPI (468/EPI) cells. The 468/EPI cells displaced enhanced resistance to EPI compared with parental cells. The cells were incubated in EPI-containing medium for 72 h. **(B)** Dose–response curve of SK-BR-3 and SK-BR-3/EPI (SK/EPI) cells. SK/EPI cells displayed enhanced resistance to EPI compared with parental cells. The cells were incubated in EPI-containing medium for 72 h. **(C,D)** P-gp and BCRP were significantly upregulated in 468/EPI **(C)** and SK/EPI **(D)** cells compared with that in parental cells, as shown by Western blot and qRT-PCR assays. **(E)** Western blot analysis of P-gp and BCRP protein expression in 468/EPI and SK/EPI cells treated with siBCRP, siP-gp, or control siRNAs. **(F)** Dose–response curves of 468/EPI and SK/EPI cells treated with siBCRP, siP-gp, or control siRNAs. The cells were incubated in EPI-containing medium for 72 h. **(G)** Western blot analysis shows that BCRP was overexpressed in MDA-468 and BT549 cells by BCRP-overexpressing lentivirus. **(H)** Dose–response curves of MDA-468 and BT549 cells with or without BCRP overexpression. The cells were incubated in EPI-containing medium for 72 h. All data are shown as the mean ± SD; **p < 0.01, ***p < 0.001, ****p < 0.0001 and ns *p* > 0.05 versus control, *N* = 3.

We established two EPI-resistant breast cancer cell lines, named SK-BR-3/EPI (SK/EPI) and MDA-MB-468/EPI (468/EPI), following a previously described procedure (Zhang et al., 2015b; Fan et al., 2019). To examine the resistance ability of the two established cell lines, we challenged the cells with increased EPI concentration and calculated the IC_50_ values. Results showed a significant elevation of IC_50_ in drug-resistant cell lines (468/EPI and SK/EPI) compared with that in the parental cell lines (MBA-468 and SK-BR-3), as shown in Figures 1A,B. As we expected, BCRP and P-gp, two well-known drug efflux pumps, were significantly upregulated in drug-resistant cells at the mRNA and protein levels (Figures 1C,D). These data demonstrated that drug resistance ability was successfully established in the breast cancer cell lines.

As BCRP and P-gp can efflux drugs across the cell membrane, we next determined whether both of them were essential to the drug-resistant phenotype of cells. Interestingly, we found that BCRP silencing mildly increased IC_50_, while P-gp silencing markedly reduced the IC_50_ of drug-resistant cells compared with that of the controls, suggesting that P-gp but not BCRP exerted a dominant effect on the efflux of EPI in these cell models (Figures 1E,F). Moreover, BCRP overexpression in drug-sensitive cells MDA-468 and BT549 exerted little resistant-promoting effect when the cells were treated with EPI (Figures 1G,H), suggesting that these cell lines may lack a favorite molecular context to support BCRP performing its drug pump activity. Taken together, these results suggested that BCRP was not the dominant drug pump that contributed to the exclusion of EPI in the two specific drug-resistant cell models that expressed multiple ABC transporters.

### Breast Cancer Resistant Protein Confers Survival Advantages in Drug-Resistant Breast Cancer Cells

**FIGURE 2:**
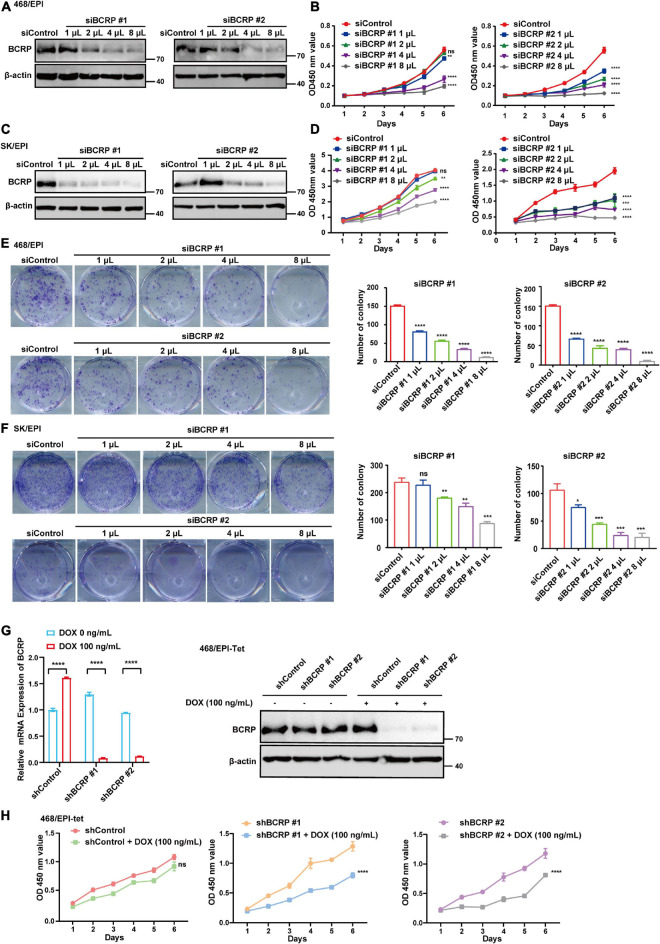
BCRP confers proliferation advantages to drug-resistant breast cancer cells. **(A)** Western blot analysis of BCRP protein expression in 468/EPI cells treated with increased amount of BCRP siRNAs. **(B)** Proliferation of 468/EPI cells treated with an increased amount of BCRP siRNAs (two-way ANOVA test). **(C)** Western blot analysis of BCRP protein expression in SK/EPI cells treated with an increased amount of siBCRP siRNAs. **(D)** Proliferation of SK/EPI cells treated with an increased amount of siBCRP siRNAs (two-way ANOVA test). For siBCRP #1, 3,000 cells were seeded, and for siBCRP#2, 1,000 cells were seeded. **(E)** Colony formation ability of 468/EPI cells treated with an increased amount of BCRP siRNAs (independent Student’s *t*-test). **(F)** Colony formation ability of SK/EPI cells treated with an increased amount of BCRP siRNAs (independent Student’s *t*-test). For siBCRP #1, 2,000 cells were seeded, and for siBCRP#2, 800 cells were seeded. **(G)** Expression of BCRP protein and mRNA in 468/EPI-tet or control cells treated with 100 ng/ml Dox, as shown by qRT-PCR and Western blot analysis (independent Student’s *t*-test). Results showed that BCRP was successfully silenced by the Dox-inducible shRNA-expressing system. **(H)** Proliferation of 468/EPI-tet or control cells treated with 100 ng/ml Dox (two-way ANOVA test). All data are shown as the mean ± SD; *p < 0.05. **p < 0.01, ***p < 0.001, ****p < 0.0001 and ns *p* > 0.05 versus control, *N* = 3.

The sustained proliferation and evasion of apoptosis are the two key factors contributing to the enhanced survival ability of drug-resistant cells. The established drug-resistant cell lines showed drastic upregulation of BCRP and P-gp compared with that in parental cells, and BCRP silencing did not sensitize these cells to EPI. Accordingly, we next investigated the drug efflux-independent functions of BCRP in drug-resistant cell lines. We found that BCRP knockdown significantly inhibited the proliferation ability of drug-resistant cells compared with that of control cells (Figures 2A–D). Similarly, colony formation ability was abolished when BCRP was silenced in drug-resistant cells (Figures 2E,F). To eliminate the potential influence caused by transfection, we established an inducible BCRP knockdown cell line by stably transfecting EZ-tet-pLKO-BCRP vector into 468/EPI cells. Knockdown efficacy was then verified by Western blot and qRT-PCR assays (Figure 2G). Consistently, we found that the knockdown of BCRP by Dox-induced shRNA significantly inhibited cell proliferation compared with control cells (Figure 2H). However, when drug-sensitive cells (MDA-468 and BT549) had BCRP overexpression, the proliferation ability was not significantly changed, suggesting that the proliferation-promoting role of BCRP specifically existed in drug-resistant cells (Supplementary Figure 1A). Collectively, these data suggested that BCRP conferred proliferation advantage on drug-resistant cells independent of its drug efflux activity.

**FIGURE 3:**
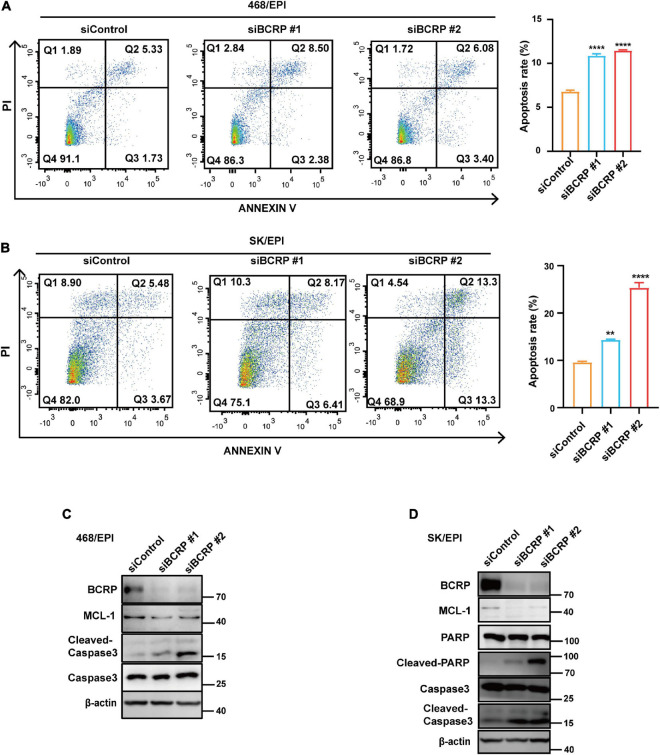
Loss of BCRP promotes the apoptosis of drug-resistant breast cancer cells. **(A,B)** Apoptosis rate of 468/EPI cells **(A)** and SK/EPI cells **(B)** with or without BCRP silencing, as measured by flow cytometry. The cells were treated with siBCRP siRNAs for 72 h and then subjected to apoptosis analysis. **(C,D)** Western blot analysis of apoptosis-related proteins in 468/EPI cells **(A)** and SK/EPI cells with or without BCRP silencing. The cells were treated with siBCRP siRNAs for 72 h, and then, the proteins were analyzed by Western blot. All data are shown as the mean ± SD; independent Student’s *t*-test, **p < 0.01, and ****p < 0.0001 versus control, *N* = 3.

Furthermore, we performed Annexin V–PI double staining to study the effect of BCRP knockdown on cell apoptosis. Notably, BCRP silencing in drug-resistant 468/EPI and SK/EPI cells induced the cells to undergo apoptosis (Figures 3A,B). The apoptosis-related protein Cleaved caspase 3 was also upregulated in BCRP-silenced drug-resistant cells compared with that in controls cells (Figures 3C,D). We also found that the antiapoptotic protein MCL1 was significantly downregulated in BCRP-silenced drug-resistant cells (Figures 3C,D). In summary, these results suggested that BCRP was vital for the drug-resistant cells to sustain their proliferation and evade programmed cell death.

### Breast Cancer Resistant Protein Inhibits Oxidative Phosphorylation in Drug-Resistant Breast Cancer Cells

**FIGURE 4:**
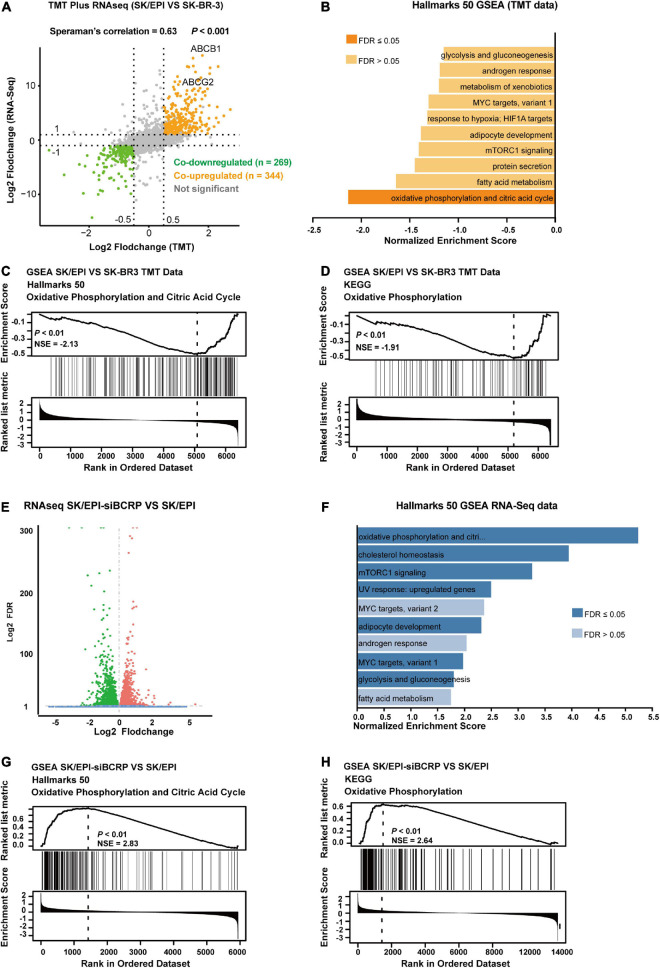
BCRP inhibits OXPHOS in drug-resistant breast cancer cells. **(A)** Nine-quadrant plot shows the differentially expressed transcripts/proteins in RNA-Seq and TMT-based proteomics. The co-upregulated and co-downregulated transcripts/proteins are shown as red dots and blue dots, respectively. **(B)** GSEA shows the top 10 downregulated signal pathways in SK/EPI cells compared with that in parental cells. **(C,D)** GSEA shows that phosphorylation- and citric acid cycle-related proteins were enriched in parental cells (SK-BR-3) compared with drug-resistant cells (SK/EPI) using Hallmarks 50 **(C)** and KEGG **(D)** gene sets. **(E)** Volcano plot shows differentially expressed genes in BCRP-silenced SK/EPI cells versus SK-BR-3 cells. **(F)** GSEA shows the top 10 upregulated signaling pathways in BCRP-silenced SK/EPI cells compared with that in control cells. **(G,H)** GSEA shows that phosphorylation- and citric acid cycle-related proteins were enriched in BCRP-silenced SK/EPI cells compared with control cells using Hallmarks 50 **(G)** and KEGG **(H)** gene sets.

To explore the function of BCRP in drug-resistant breast cancer cells in-depth, we analyzed the mRNA and protein expression of drug-resistant cells (SK/EPI) and the matched parental cells (SK-BR-3) by RNA-seq and TMT-based high-throughput proteomics. We found that the differentially expressed mRNA and proteins in SK/EPI versus SK-BR-3 cells were highly consistent (Figure 4A and Supplementary Table 3). We further performed GSEA using TMT-based proteomic data, which showed that several metabolic-related pathways were downregulated in the resistant cells (Figure 4B). Notably, GSEA showed that the OXPHOS- and citric acid cycle-related signaling pathways were deactivated in drug-resistant SK/EPI cells (Figures 4C,D). The detailed results of proteomics and RNA-seq data for SK/EPI and SK-BR-3 cells are shown in Supplementary Table 3.

We further examined the transcriptome-wide alteration of SK/EPI cells upon BCRP silencing. Bioinformatics analysis suggested that 2,492 genes were downregulated and 2,837 genes were upregulated in SK/EPI cells versus SK-BR-3 cells (Figure 4E). Notably, a large number of metabolic-related signaling pathways were upregulated in BCRP-silenced SK/EPI cells compared with that in control cells, as suggested by GSEA (Figure 4F). GSEA also suggested that OXPHOS- and citric acid cycle-related signaling pathways were activated upon BCRP silencing in SK/EPI cells (Figures 4G,H). The detailed results of RNA-seq data for BCRP-silenced SK/EPI and Nc-silenced SK/EPI cells are shown in Supplementary Table 4.

Additionally, we used clustered heatmap to examine the expression levels of several selected OXPHOS-related genes in SK-BR-3, SK/EPI, and BCRP-silenced SK/EPI cells. Consistent with GSEA data, these OXPHOS-related genes were significantly downregulated in SK/EPI cells compared with that in the SK-BR-3 cells (Supplementary Figure 2A). Interestingly, when BCRP was silenced in SK/EPI cells, these genes were re-expressed compared with those in control cells (Supplementary Figure 2B). However, BCRP overexpression in drug-sensitive cells did not alter the expression of these selected genes, indicating the function of BCRP in OXPHOS was specifically in the drug-resistant cells (Supplementary Figures 2C,D). Collectively, these *in silico* data were consistent with the extensively accepted perspective that drug-resistant cells rely more on anaerobic than on aerobic respiration to sustain their energy need. Notably, BCRP silencing reactivated the suppressed OXPHOS in drug-resistant cells, suggesting that BCRP was involved in regulating the OXPHOS-related signaling pathway in drug-resistant breast cancer cells.

### Breast Cancer Resistant Protein Is Localized in Mitochondria of Drug-Resistant Breast Cancer Cells

**FIGURE 5:**
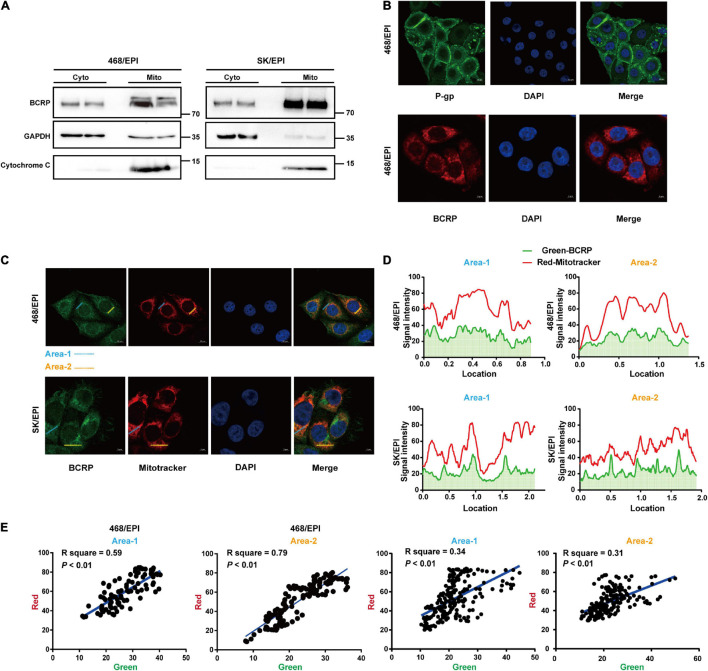
BCRP is localized in the mitochondria of drug-resistant breast cancer cells. **(A)** Western blot analysis shows BCRP was expressed in the mitochondria of drug-resistant cells. The mitochondrial and cytoplasmic proteins were isolated and analyzed using a Mitochondrial Extraction Kit; GAPDH and Cytochrome C served as markers for cytoplasm and mitochondria, respectively. **(B)** IF shows the subcellular localization of P-gp and BCRP in 468/EPI cells. **(C)** IF shows that BCRP was localized in the mitochondria of drug-resistant cells. **(D)** Analysis of the fluorescent signal intensity of the area indicated in the IF image. **(E)** Correlation analysis of the signal intensity of BCRP (green) and mitochondrial (red) through linearized regression. The signal intensity for each fluorescent channel was analyzed by ImageJ, and the linear correlation coefficients were calculated.

Bioinformatics analyses indicated that BCRP was linked with OXPHOS, which primarily occurred inside mitochondria, so we further analyzed the subcellular localization of BCRP in drug-resistant cells. The cytosolic and mitochondrial fraction of two drug-resistant cell lines, SK/EPI and 468/EPI, were isolated and analyzed by Western blot. Results showed that BCRP was expressed in the mitochondria fraction of these cells (Figure 5A). Notably, IF analysis showed that BCRP was primarily localized in the cytoplasm instead of the cell membrane in drug-resistant cells, whereas P-gp was dominantly expressed in the cell membrane, further supporting the notion that BCRP was not the major drug efflux pump that excluded the chemotherapy drugs in these two specific drug-resistant cell models (Figure 5B).

Bioinformatics analysis indicated BCRP was linked with OXPHOS, which primarily occurred in cell mitochondria. We next determined the subcellular localization of BCRP in drug-resistant cells. Importantly, IF analysis showed that the signals of BCRP and mitochondria overlapped (Figure 5C). Furthermore, we analyzed the signaling intensity of BCRP and MitoTracker in drug-resistant cells (Figure 5D), and the correlation analysis strongly suggested BCRP and mitochondria were colocalized in drug-resistant cells (Figure 5E). Collectively, these data suggested that mitochondria BCRP may involve the regulation of the OXPHOS of drug-resistant breast cancer cells.

### Breast Cancer Resistant Protein Inhibits Reactive Oxygen Species Production in Drug-Resistant Breast Cancer Cells

**FIGURE 6:**
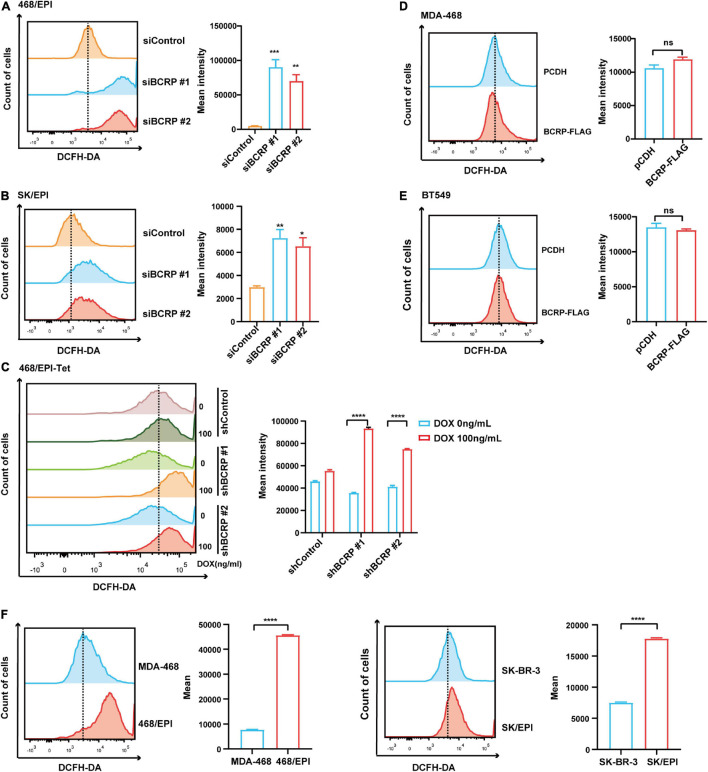
BCRP inhibits ROS production in drug-resistant breast cancer cells. **(A,B)** Analysis of intracellular ROS level in 468/EPI cells **(A)** and SK/EPI cells **(B)** treated with siBCRP or control siRNAs. The cells were treated with the indicated siRNAs for 48 h, and then, the ROS level was determined. **(C,D)** Analysis of intracellular ROS level in MDA-468 cells **(C)** and BT549 cells **(D)** with or without BCRP overexpression. **(E)** Analysis of intracellular ROS level in 468/EPI-tet or control cells with or without Dox treatment. The cells were treated with the Dox for 48 h, and then, the ROS level was determined. **(F)** Analysis of intracellular ROS level in drug-resistant cells (468/EPI and SK/EPI) and the matched parental cells (MDA-468 and SK-BR-3). All data are shown as the mean ± SD; *p < 0.05. **p < 0.01, ***p < 0.001, ****p < 0.0001, and ns *p* > 0.05 versus control, *N* = 3.

Mitochondria is the major source of intercellular ROS. We have demonstrated that BCRP was localized in mitochondria and potentially linked with mitochondrial activity. To investigate the connection between BCRP and ROS production, BCRP was knocked down in the drug-resistant SK/EPI and 468/EPI cells by using two independent siRNAs, and the intercellular ROS level was measured by using DCFH-DA ROS probe. Results showed that intercellular ROS level was significantly elevated in BCRP-silenced SK/EPI and 468/EPI cells compared with that in control cells (Figures 6A,B). Furthermore, BCRP was silenced in the Dox-inducible shBCRP cell line (468/EPI-tet) by treating the cells with 100 ng/ml Dox. Consistently, ROS level was profoundly elevated in Dox-treated cells compared with that in control cells (Figure 6C).

Next, we forced expressed BCRP in drug-sensitive cells (MDA-468 and SK-BR-3) and determined the ROS level. However, we did not observe a significant change in ROS level in BCRP-overexpressed cells compared with control cells (Figures 6D,E), suggesting BCRP was not linked with intercellular ROS production in drug-sensitive breast cancer cells. We also found that the baseline ROS level in drug-resistant cells was significantly higher than that in parental cells (Figure 6F), indicating that drug-resistant cells were more vulnerable to ROS-related damage when the intracellular ROS level was further elevated. In summary, these results indicated that BCRP was an essential factor that maintained the homeostasis of intercellular ROS in drug-resistant cells. The elevated ROS in BCRP-silenced cells may exceed the safety threshold and induce the cells to undergo apoptosis.

### Knockdown of Breast Cancer Resistant Protein Inhibits the Proliferation of Drug-Resistant Breast Cancer Cells *in vivo*

**FIGURE 7:**
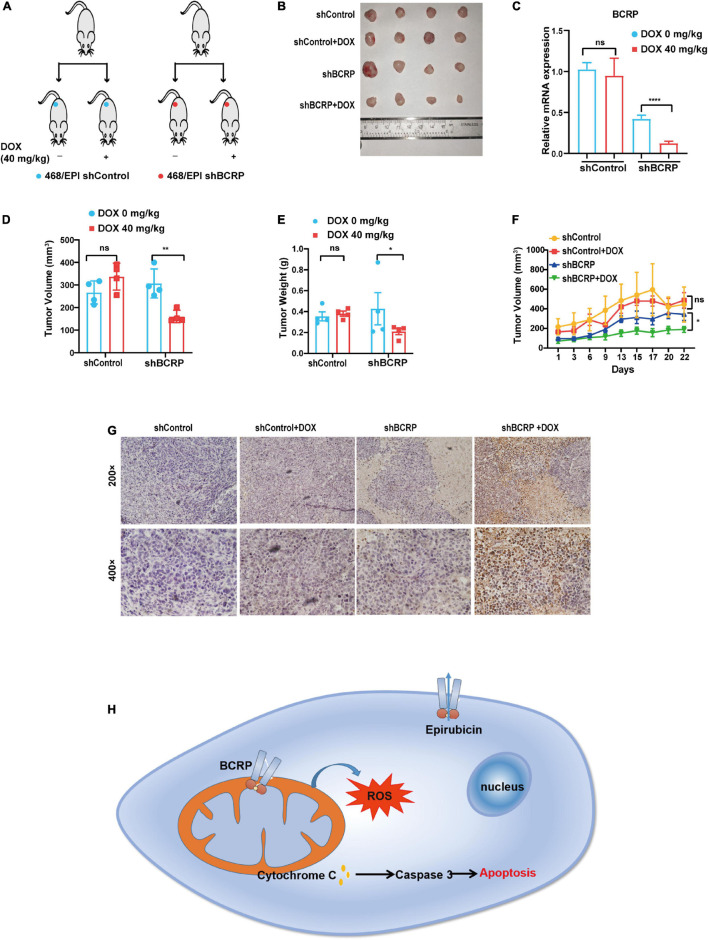
Knockdown of BCRP inhibits the proliferation of drug-resistant breast cancer cells *in vivo*. **(A)** Schematic depicting the overall design of *in vitro* experiments. **(B)** Representative image of tumors from mice receiving indicated treatments. **(C)** Expression level of BCRP mRNA in xenograft tumors in mice receiving indicated treatments (independent Student’s *t*-test). **(D)** Volume of tumors in mice receiving indicating treatments (independent Student’s *t*-test). **(E)** Weight of tumors in mice receiving indicated treatments (independent Student’s *t*-test). **(F)** Growth curves of inoculated tumors in mice receiving indicated treatment (two-way ANOVA test). **(G)** Representative images of TUNEL staining of dissected tumors in mice receiving indicated treatment. **(H)** Schematic of the proposed molecular mechanism of this study. The mitochondrial BCRP inhibits OXPHOS to reduce the intracellular ROS production, thereby inhibiting the apoptosis of drug-resistant breast cancer cells. All data are shown as the mean ± SD; *p < 0.05. **p < 0.01, ****p < 0.0001, and ns *p* > 0.05 versus control, *N* = 4 for the *in vivo* experiments.

To investigate the biological function of BCRP in drug-resistant breast cancer cells *in vivo*, the Dox-inducible BCRP knockdown cells (468/EPI-shBCRP-tet) and the control cells (468/EPI-shControl-tet) were inoculated into female nude mice subcutaneously. Fourteen days after inoculation, the mice were given 40 mg/kg Dox or water every 2 days by gavage, and the tumor volume was continuously monitored (Figures 7A,B). The tumors were dissected 25 days after gavage, and qRT-PCR demonstrated that BCRP was successfully silenced in the tumor formed by 468/EPI-tet-shBCRP cells, in which the host mice received Dox gavage (Figure 7C). Importantly, we found that the tumors formed by 468/EPI-shBCRP-tet cells with Dox-induced BCRP silencing showed a significant decrease in volume and weight compared with that of control tumors (Figures 7D,E). Consistently, the growth of tumors formed by drug-resistant cells (468/EPI-shBCRP-tet) was also profoundly inhibited by Dox-induced BCRP silencing (Figure 7F). Moreover, TUNEL staining showed that the tumors formed by BCRP-silenced 468/EPI cells had a strong positive signal, suggesting that these cells underwent extensive apoptosis upon Dox-induced BCRP knockdown (Figure 7G). Collectively, these data indicated that BCRP was an important factor promoting the progression of drug-resistant cells by inhibiting apoptosis *in vivo*.

## Discussion

Chemotherapy is the most commonly used treatment method of managing breast cancer. However, as cancer cells evolve to adapting the drug cytotoxicity, the drug-resistant phenotype is eventually acquired, leading to treatment failure (Kathawala et al., 2015; Fletcher et al., 2016). ABC transporters are key contributors to the establishment of multidrug resistance (MDR), a phenomenon that aids survival of cancer cells in the presence of anticancer drugs, even in drugs that cells never encounter (Kathawala et al., 2015). By positively transporting drugs across the plasma membrane, ABC transporters maintain a relatively low intracellular drug load, causing cancer cells to acquire a strong ability to counteract drug-induced toxicity (Kathawala et al., 2015; Robey et al., 2018). Intriguingly, recent studies have suggested that ABC transporters have important biological functions beyond MDR. Several reports have pointed out that the dysregulation of ABC transporters is connected with aggressive phenotypes of cancers, e.g., invasion and metastasis, evasion of apoptosis, sustained proliferation, and the emergence of cancer stem cells (Begicevic and Falasca, 2017; Muriithi et al., 2020). ABCC1, ABCC3, and ABCC4 have been suggested to be linked with tumor growth and prognosis in neuroblastoma (Porro et al., 2010; Westover and Li, 2015). In breast cancer, ABCC11 overexpression has been observed in tumors with a highly aggressive molecular subtype (Yamada et al., 2013). High expression of ABCC1 and low expression of ABCC8 are correlated with the aggressiveness of breast cancer (Hlavac et al., 2013). However, most of these studies are correlational, so an in-depth analysis of the related molecular mechanism is needed. Herein, we discovered a novel role of BCRP in promoting the survival of drug-resistant breast cancer cells by regulating intracellular ROS level. We revealed that BCRP was indeed localized in mitochondria but not in the cell membrane in certain drug-resistant cell models. BCRP downregulation significantly inhibited proliferation and induced apoptosis in resistant cells, suggesting a novel role of BCRP in cancer cells beyond the MDR.

BCRP upregulation is associated with the progression of various tumors. The drug pump activity is the best-characterized mechanism that is responsible for the BCRP-induced drug resistance. For instance, BCRP silencing induces cell cycle arrest, thereby inhibiting proliferation in DU145, MCF7, and A549 cells (Chen et al., 2010). BCRP can also be translocated into the nucleus by interacting with *CDH1* promoter to regulate metastasis in lung cancer cells (Liang et al., 2015). However, the MDR-independent roles of BCRP in drug-resistant breast cancer cells have not yet been characterized. In the process of drug-resistant establishment, cancer cells go through a series of genetic and metabolic reprogramming, conferring the cells with a strong ability to manage intracellular redox homeostasis and thus enhance their survival capability. In the present study, we identified a novel role of BCRP in protecting drug-resistant breast cancer cells from apoptosis by regulating intracellular ROS level. In contrast to P-gp, BCRP was primarily localized in the cytoplasm instead of the cell membrane, indicating that the drug efflux activity was not essential in our MDR cell models. However, BCRP silencing drastically impaired the cell proliferation ability. The apoptosis of drug-resistant cells was also more evident upon BCRP knockdown. These results indicated that BCRP was pivotal to the survival of drug-resistant cells, even in a favorable environment without drug-induced cytotoxicity. Interestingly, the ectopic expression of BCRP in drug-sensitive breast cancer cells showed no influence on cell proliferation and, more apparently, on drug resistance capabilities. This finding may be due to the lack of a specific molecular environment essential for BCRP to perform its normal transport function in sensitive cells. It also suggested that the function of BCRP was highly cell type dependent.

Emerging evidence suggests that ROS involves various cellular processes, such as proliferation, apoptosis, gene mutation, and signaling transduction in cells (Cui et al., 2018; Sies and Jones, 2020). ROS are generally believed to act as a “double-edged sword” involved in various biological processes in cells. A low level of intracellular ROS can act as a multigene to promote the cell proliferation and survival of cancer cells, whereas an intermediate level of ROS can cause cell cycle arrest (Yang et al., 2018; Sies and Jones, 2020). However, a high level of ROS damages macromolecules in cells such as DNA, RNA, plasma membranes, and proteins, eventually inducing cells to undergo irreversible apoptosis (Cui et al., 2018). Some anticancer drugs, such as EPI, kill cancer cells partially by elevating intracellular ROS level (Lo and Wang, 2013; Yang et al., 2018). Thus, acquiring a robust anti-ROS ability is essential for drug-resistant cancer cells to survive in harsh environments. Interestingly, we found that drug-resistant cells had a higher baseline ROS level than parental cells. This finding may be explained by drug-resistant cells adopting well-coordinated mechanisms that maintain the intracellular ROS at a favorite level to make this “double-edged sword” tend toward the beneficial side. Hence, BCRP may act as a regulator that controls and maintains an onco-promoting intracellular redox environment to facilitate the survival and advance of drug-resistant cells.

In mitochondria, ROS are primarily generated from leaked electrons in the electron transport chain, which is tightly linked with the OXPHOS rate in the cells. In our cell models, we also found OXPHOS-related signaling pathways were deactivated in drug-resistant cells compared with that in parental cells, consistent with the well-recognized feature that drug-resistant cells have decreased aerobic aspiration (Bhattacharya et al., 2016; Icard et al., 2018). The relatively low level of OXPHOS may protect cells from excessive ROS poisoning. Drug-resistant cells have higher ROS level than parental cells, so the adjustable space for ROS is limited. Thus, drug-resistant cells may be more vulnerable to ROS fluctuations. Interestingly, bioinformatics analysis results suggested that BCRP silencing reactivated the OXPHOS-related signaling pathways, which may cause increased ROS level and may thus eventually push the cells beyond their ROS-tolerant threshold. Importantly, BCRP was localized in the mitochondria in drug-resistant cells. Considering that the mitochondria are the primary source of ROS production, we speculated that mitochondrial BCRP may partially contribute to the inhibition of OXPHOS-related ROS production, thereby preventing the cells from ROS overload. However, the detailed molecular foundation linking BCRP and OXPHOS remains to be elucidated.

In summary, we identified a novel, drug efflux-independent function of BCRP in drug-resistant breast cancer cells. The elevated BCRP, instead of pumping the drug out of the cells, may act as a modulator to inhibit mitochondrion-originating ROS, thereby preventing cells from drug-induced oxidative toxicity and apoptosis (Figure 7H). This work demonstrated that BCRP is essential in the proliferation and survival of drug-resistant breast cancer cells.

## Data Availability Statement

The datasets presented in this study can be found in online repositories. The names of the repository/repositories and accession number(s) can be found below: https://www.ncbi.nlm.nih.gov/geo/ under GSE181632.

## Ethics Statement

The animal study was reviewed and approved by the Animal Ethics Committee of Tianjin Medical University Cancer Institute and Hospital.

## Author Contributions

HZ, FZ, and RN: conception and design, study supervision. HZ, XH, ZSW, ZYW, YC, RT, YZ, BH, HL, SR, XZ, and JT: acquiring of data. HZ, XH, RN, and FZ: writing – review and revision of the manuscript. All authors contributed to the article and approved the submitted version.

## Conflict of Interest

The authors declare that the research was conducted in the absence of any commercial or financial relationships that could be construed as a potential conflict of interest.

## Publisher’s Note

All claims expressed in this article are solely those of the authors and do not necessarily represent those of their affiliated organizations, or those of the publisher, the editors and the reviewers. Any product that may be evaluated in this article, or claim that may be made by its manufacturer, is not guaranteed or endorsed by the publisher.

## Footnotes

1
http://www.webgestalt.org/
