# Supplementary material for: Mitochondrial Breast Cancer Resistant Protein Sustains the Proliferation and Survival of Drug-Resistant Breast Cancer Cells by Regulating Intracellular Reactive Oxygen Species
Source: Front Cell Dev Biol. 2021 Sep 28;9:719209. doi: 10.3389/fcell.2021.719209 (PMC8505676; doi:10.3389/fcell.2021.719209)
Supplement: Supplementary file 1 [file Table_1.DOCX]

**Supplemental Table S1. Antibodies used in this study**

| Target | Species | Dilution (Application) | Manufacture |
| --- | --- | --- | --- |
| P-gp | Rabbit | 1:1000 (WB)  1:200 (IF) | Santa Cruz Biotechnology sc-55510  Santa Cruz Biotechnology sc-55510-AF488 |
| BCRP | Mouse | 1:500 (WB)  1:200 (IF) | Santa Cruz Biotechnology sc-377176  Santa Cruz Biotechnology sc-377176-AF647 |
| MCL-1 | Rabbit | 1:1000 (WB) | Santa Cruz Biotechnology sc-74436 |
| Caspase 3 | Rabbit | 1:1000 (WB) | Cell Signaling Technology 9662S |
| Cleaved-caspase 3 | Rabbit | 1:500 (WB) | Cell Signaling Technology 9664S |
| PARP | Rabbit | 1:1000 (WB) | Cell Signaling Technology 9532S |
| Cleaved-PARP | Rabbit | 1:500 (WB) | Cell Signaling Technology 5625S |
| β-actin | Mouse | 1:3000 (WB) | Sigma Aldrich A1978 |
| GAPDH | Mouse | 1:3000 (WB) | Santa Cruz Biotechnology sc-47724 |
| Cytochrome c | Mouse | 1:1000 (WB) | Santa Cruz Biotechnology sc-13156 |
| HRP-linked Anti-Rabbit IgG | Goat | 1:4000 (WB) | BIO-RAD #1706515 |
| HRP-linked Anti-Mouse IgG | Goat | 1:4000 (WB) | BIO-RAD #1706516 |

WB, Western blot; IF, Immunofluorescent stain
