# Supplementary material for: Mitochondrial Breast Cancer Resistant Protein Sustains the Proliferation and Survival of Drug-Resistant Breast Cancer Cells by Regulating Intracellular Reactive Oxygen Species
Source: Front Cell Dev Biol. 2021 Sep 28;9:719209. doi: 10.3389/fcell.2021.719209 (PMC8505676; doi:10.3389/fcell.2021.719209)
Supplement: Supplementary file 5 [file Data_Sheet_1.docx]

**Supplementary Figure Legends**

**Supplementary Figure S1. BCRP did not affect the proliferation of drug-sensitive cells.**

**(A)** Cell counting kits 8 assay shows that BCRP overexpression in drug-sensitive MDA-468 and BT-549 cells did not influence cell proliferation. All data are shown as the mean ± SD, ns *P* > 0.05 versus control, N=3.

**Supplementary Figure S2. Expression levels of oxidative phosphorylation-related genes in SK-BR-3, SK/EPI and BCRP-silenced SK/-EPI cells.**

**(A)** Heatmap shows the selected oxidative phosphorylation (OXPHOS)-related genes in SK-BR-3 and SK/EPI cells. **(B)** Heatmap shows the selected OXPHOS-related genes in SK/EPI and BCRP-silenced SK/EPI cells. **(C, D)** Expression levels of the selected OXPHOS-related genes were unaffected by BCRP overexpression in drug-sensitive MDA-468 and BT549 cells. All data are shown as the mean ± SD, *** *P* < 0.001 and ns *P* > 0.05 versus control, N=3.
