# Supplementary figures and images for: Mitochondrial Breast Cancer Resistant Protein Sustains the Proliferation and Survival of Drug-Resistant Breast Cancer Cells by Regulating Intracellular Reactive Oxygen Species
Source: Front Cell Dev Biol. 2021 Sep 28;9:719209. doi: 10.3389/fcell.2021.719209 (PMC8505676; doi:10.3389/fcell.2021.719209)

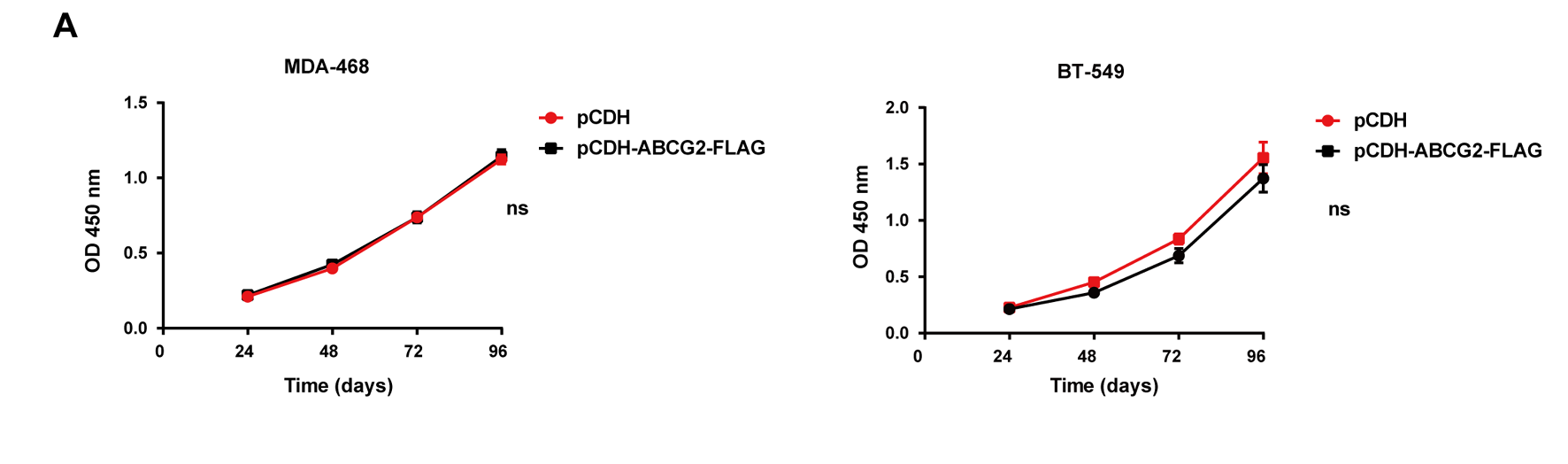

Supplement: Supplementary file 6 [file Image_1.TIF]

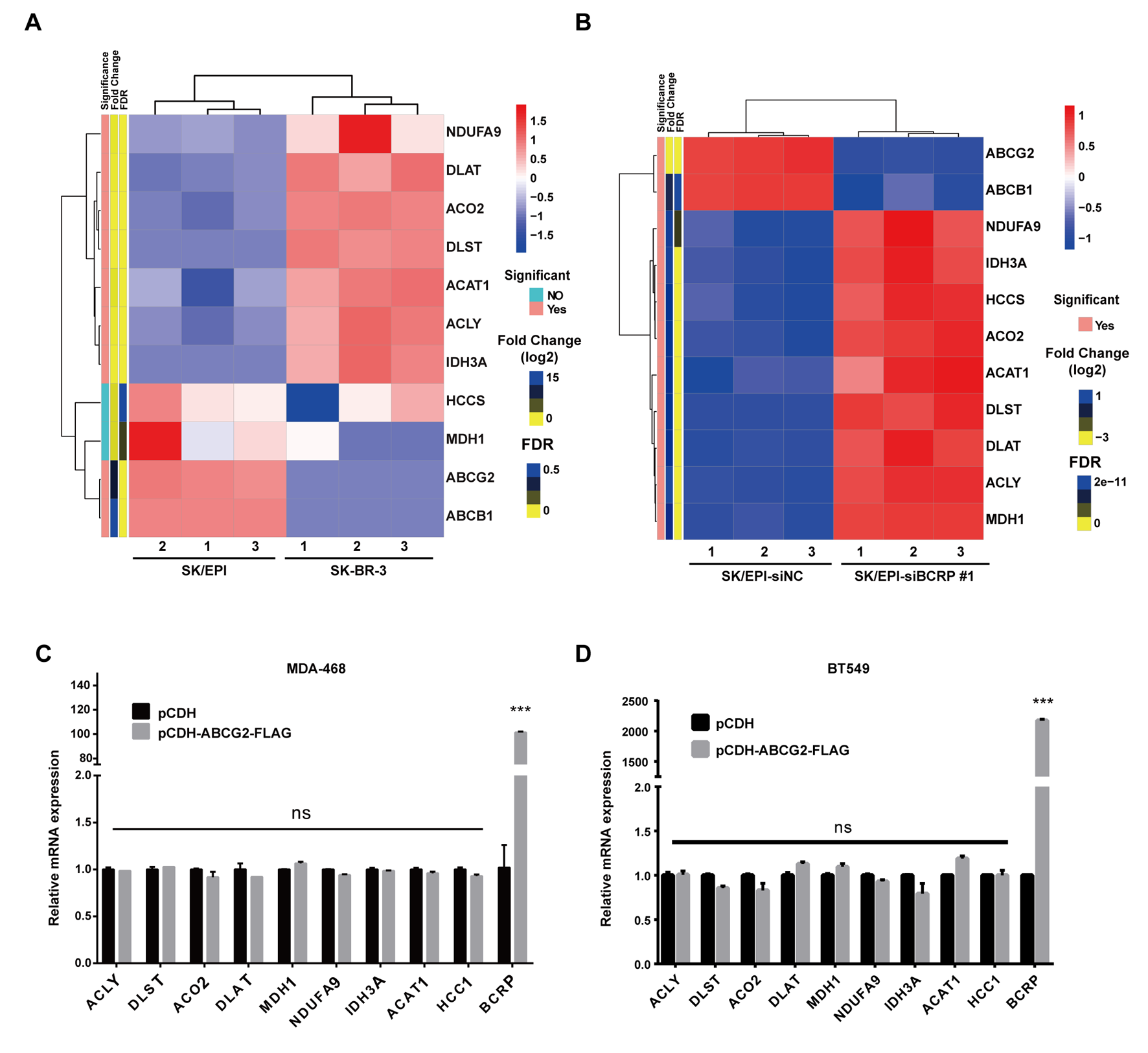

Supplement: Supplementary file 7 [file Image_2.TIF]
